# Cloning of the *Zygosaccharomyces bailii GAS*1 homologue and effect of cell wall engineering on protein secretory phenotype

**DOI:** 10.1186/1475-2859-9-7

**Published:** 2010-01-26

**Authors:** Simone Passolunghi, Luca Riboldi, Laura Dato, Danilo Porro, Paola Branduardi

**Affiliations:** 1 University of Milano-Bicocca, Department of Biotechnology and Bioscience, Milan, Italy; 2 CPC Biotech S.r.l., Naples, Italy

## Abstract

**Background:**

*Zygosaccharomyces bailii *is a diploid budding yeast still poorly characterized, but widely recognised as tolerant to several stresses, most of which related to industrial processes of production. Because of that, it would be very interesting to develop its ability as a cell factory. Gas1p is a β-1,3-glucanosyltransglycosylase which plays an important role in cell wall construction and in determining its permeability. Cell wall defective mutants of *Saccharomyces cerevisiae *and *Pichia pastoris*, deleted in the *GAS*1 gene, were reported as super-secretive. The aim of this study was the cloning and deletion of the *GAS*1 homologue of *Z. bailii *and the evaluation of its deletion on recombinant protein secretion.

**Results:**

The *GAS*1 homologue of *Z. bailii *was cloned by PCR, and when expressed in a *S. cerevisiae GAS*1 null mutant was able to restore the parental phenotype. The respective *Z. bailii* Δ*gas*1 deleted strain was obtained by targeted deletion of both alleles of the *ZbGAS*1 gene with deletion cassettes having flanking regions of ~400 bp. The morphological and physiological characterization of the *Z. bailii *null mutant resulted very similar to that of the corresponding *S. cerevisiae *mutant. As for *S. cerevisiae*, in the *Z. bailii *Δ*gas*1 the total amount of protein released in the medium was significantly higher. Moreover, three different heterologous proteins were expressed and secreted in said mutant. The amount of enzymatic activity found in the medium was almost doubled in the case of the *Candida rugosa *lipase CRL1 and of the *Yarrowia lipolytica *protease XPR2, while for human IL-1β secretion disruption had no relevant effect.

**Conclusions:**

The data presented confirm that the engineering of the cell wall is an effective way to improve protein secretion in yeast. They also confirmed that *Z. bailii *is an interesting candidate, despite the knowledge of its genome and the tools for its manipulation still need to be improved. However, as already widely reported in literature, our data confirmed that an "always working" solution to the problems related to recombinant protein production can be hardly, if never, found; instead, manipulations have to be finely tuned for each specific product and/or combination of host cell and product.

## Background

The "non-conventional" yeast *Zygosaccharomyces bailii *has been proposed as a possible new player in biotech processes arena [1]. Its tolerance to high sugar concentrations, low pH environments caused by organic acids, relatively high temperatures [2] could allow its growth under restrictive conditions. These features can be very useful for industrial fermentations because they can simplify the process, improving the economical value. In addition, the high specific growth rate of *Z. bailii *and its high biomass yield render this yeast particularly attractive for those applications were the biomass itself, or a specific protein or metabolite, is the desired product. In respect to recombinant protein production, it has been demonstrated that production and secretion levels of the recombinant human interleuchin-1β (rhIL-1β) and of fungal glucoamylases in *Z. bailii *are higher than the levels obtained with *S. cerevisiae *by the use of the same centromeric expression vector [3].

From an industrial point of view, production and secretion capabilities of the host systems currently in use are still insufficient. In particular, secretion of the heterologous protein often represents one of the main bottlenecks. Different strategies can be utilized in this respect, and among them are: improvement of the heterologous gene expression levels, signal sequence optimization, co-expression of chaperones and foldases, introduction of mutations which improve secretion capabilities and reduction of the proteolytic activity in secretion vesicles [4].

Since the yeast cell wall constitutes a physical barrier to large molecules transit, it represents one of the main limits for heterologous protein secretion. Thus, mutations which directly or indirectly alter the cell wall organization can lead to better secretion capabilities of a yeast host [5].

Mutations in genes involved in the construction and in the maintenance of the cell wall, such as *PMR*1, *SEC*14, *ERD*1, *MNN*9 and *MNN*10, have in some cases been demonstrated to lead to supersecretive mutants in *S. cerevisiae *and other yeasts [5]. The results evidence a tight correlation between glycosylation processes and protein secretion [6]. It was hypothesized that this correlation is not directly linked to the heterologous secreted proteins, but rather to an altered structure of the glycosidic residues added to the cell wall (glycol)proteins [5], which in turn leads to an altered permeability of the cell wall itself.

Among the already mentioned target, also the inactivation of the *GAS*1 gene, whose product is directly involved in the synthesis of the cell wall, led to a hypersecretive phenotype in *S. cerevisiae *[7]. The *Sc*Gas1p is an extracellular glycoprotein, anchored to the cell membrane through a GPI tail, which has a β-1,3-glucanosyltransferase activity. This enzyme plays a central role in the formation of cell wall glucans: it catalyzes the crosslinking of the glucans resulting in the β-1,3-glucan elongation [8]. The identification of Gas1p homologues in other yeast species and some pathogenic fungi led to the definition of a new glycosyl-hydrolases family. Recently, the hypothetical 3D structure of the catalytic domain of the protein was built by homology modelling [9]. The *GAS*1 gene disruption leads to peculiar morphological and physiological phenotypes due to an altered cell wall structure and composition. In particular, it was reported an altered distribution of the percentages of β-1,3- and β-1,6-glucans, the latter being higher than in wild type strain [10]. It was also reported an increase in the chitin and mannoproteins levels, together with altered linkages between the different components [11]. As a direct or indirect result of these cell wall structure modifications, the *gas1 *mutant shows higher secretion levels if compared to the wild type, both for total and for the heterologous recombinant proteins, as the human insuline-like growth factor (rhIGF1), [7]. More recently, the *Pichia pastoris GAS*1 homologous gene was cloned and then deleted, also resulting in a yeast mutant showing a supersecretive phenotype for some heterologous proteins, like a yeast lipase, but not for other, like the human trypsinogen [12].

Here we demonstrate that the deletion of the *Z. bailii GAS*1 homologue leads to an improved heterologous protein secretion in this new promising host. For reaching this goal, the *ZbGAS*1 gene was firstly cloned and then deleted thanks to the setting up of a protocol for gene deletion in this non-conventional diploid yeast. We describe here the morphological and physiological characterization of the *Zb*Δ*gas*1, which, in analogy to the *Sc*Δ*gas*1 and *Pp*Δ*gas*1 mutants, resulted to have a supersecretive phenotype.

## Results

### PCR cloning for *Z. bailii GAS*1 homologue

Because of the high phylogenetic correlation between *Z. bailii *and *S. cerevisiae *[13] and because of the high percentage of identity among the few known *Z. bailii *and the corresponding *S. cerevisiae *gene sequences, our first attempt was to clone the gene homologous to *GAS*1 in *Z. bailii *by PCR amplification, using primers designed on the 3' and 5' ends of the *ScGAS*1 coding sequence, but with no success.

**Figure 1:**
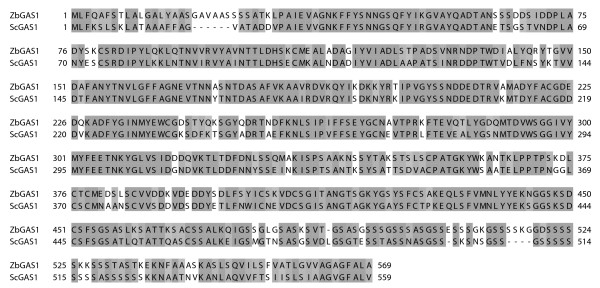
**Clustalw_aa.pdf**. Putative aminoacidic sequence of the *Zb*Gas1p protein and alignment with the homologue *Sc*Gas1p. Grey scale coloring represents aminoacid similarity based on Blosum 62 scores. Alignment was performed with ClustalW software http://www.ebi.ac.uk/clustalw and graphical editing was made using Jalview software [31].

It was therefore necessary to design internal primers choosing the most conserved regions of the gene, based on multiple sequence alignments. The *Candida glabrata *and the *Schyzosaccharomyces pombe *homologous genes (overall sequence identity to *ScGAS*1: 71% and 62% respectively), in addition to the *S. cerevisiae *one, were chosen for comparison. Two amplification primers were designed on *ScGAS*1 in (sub)regions sharing nearly 100% sequence identity with the two homologues, at about 200 bp and 300 bp from the ATG and the STOP codon, respectively. A 1137 bp fragment was amplified, using *Z. bailii *genomic DNA as a template, sharing 72% sequence identity with the corresponding internal region of *ScGAS*1. Translation into the putative aminoacidic sequence led to the identification of a region with 74% identity to the *Sc*Gas1p protein fragment spanning from the catalytic domain to the beginning of the Cys Box (WU-Blast2 alignment results, Figure 1). This confirms data reported in literature which indicate the catalytic domain and the Serine and Cysteine Boxes as the most conserved regions of the *GAS*1 homologues in different yeast species [8].

The 5' and 3' regions of the *ZbGAS*1 homologue were subsequently amplified from a Genome Walker library constructed from the *Z. bailii *genomic DNA using the Universal GenomeWalker^® ^Kit (Clontech). Gene-specific primers used were *ZbGAS1*intREV and *ZbGAS1*intFW for the 5' and 3' ends respectively (see Methods). The complete ORF of *ZbGAS*1 was reconstructed in this way, and additional 262 bp upstream to the ATG and 177 bp after the STOP codon were also sequenced (GeneBank Accession N° GU136594).

Based on the new sequence, it was possible to design specific primers for the *ZbGAS*1 gene (see Methods) and a DNA fragment of the expected length was amplified from the *Z. bailii *genomic DNA.

The complete *ZbGAS*1 ORF resulted to be 1710 bp long (+ 30 bp *vs ScGAS*1), sharing a 67% sequence identity with the *S. cerevisiae *homologue (additional file 1). This sequence encodes for a putative aminoacidic sequence of 569 aa (+10 aminoacids *vs Sc*Gas1p) having 68% identity with the *Sc*Gas1p protein (Figure 1). Sequence alignments were also performed between the newly isolated *Z. bailii *ORF and sequences of other homologous *GAS *genes in *S. cerevisiae *(*GAS*1-5): the lower identity scores obtained for sequences other than *ScGAS*1 (not shown) further indicated that we cloned the *GAS*1 homologue of *Z. bailii*.

Hence, considering the sequence similarities between the *ScGAS*1 gene and their homologues in other yeasts, the *GAS*1 gene resulted to be highly conserved also in *Z. bailii*. The overall deduced protein structure seems to be also conserved.

The *ZbGAS*1 gene is one of the few *Z. bailii *gene sequences isolated to date, hence representing a further little step towards the knowledge of this still poorly characterized yeast.

### *ZbGAS*1 gene disruption

**Figure 2:**
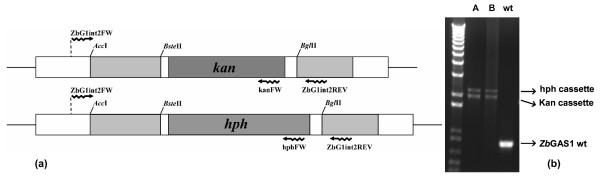
**Deletion of *ZbGAS*1**. (a) Schematic representation of the *ZbGAS*1 deletion cassettes. Restriction enzymes used to obtain the constructs and PCR primers used to verify recombination are indicated, light grey bars represents *ZbGAS*1 sequence. (b) Control PCR performed to verify the deletion of both the allelic copies of *ZbGAS*1. Two independent mutant clones (A and B) were tested. In both clones it is possible to see the presence of bands corresponding to the two deletion cassettes and the absence of the band corresponding to the wild type gene copy.

Two different *ZbGAS*1 deletion cassettes were constructed based on the *kan*^R ^and *hph*^R ^markers. Differently from the case of the *ZbYME*2 gene deletion [14], where flanking regions of about 60 to 80 bp were described to be sufficient for obtaining homologous recombination, long homologous flanking regions were necessary to obtain gene replacement. In particular, we constructed both the deletion cassettes, represented in Figure 2a, with flanking regions of about 400 bp, which resulted to be sufficient to disrupt the first and as well the second allelic copy of *ZbGAS*1, despite with quite low efficiency (about only 10% of the transformants resulted to effectively have both the deleted alleles, confirming what previously described [14]). The two cassettes (see Methods) were therefore used to transform *Z. bailii *cells, resulting in the *gas1 *null mutant. The insertion of both deletion cassettes in the homologous loci was confirmed by PCR (Figure 2b). We applied the same strategy for the deletion of the first essential *Z. bailii *gene, leading to the first auxotrophic mutant of this yeast, and in this case we had to further extend the length of the homologous region for specific recombination, and despite that the percentage of positive events was even lower (manuscript in preparation). These results could lead to hypothesize that for targeted gene deletion in *Z. bailii *the length of the flanking regions could be crucial according to the specific role that a specific protein plays, thus explaining results obtained by other researchers [14].

### Morphological and physiological characterization of the null mutant

**Figure 3:**
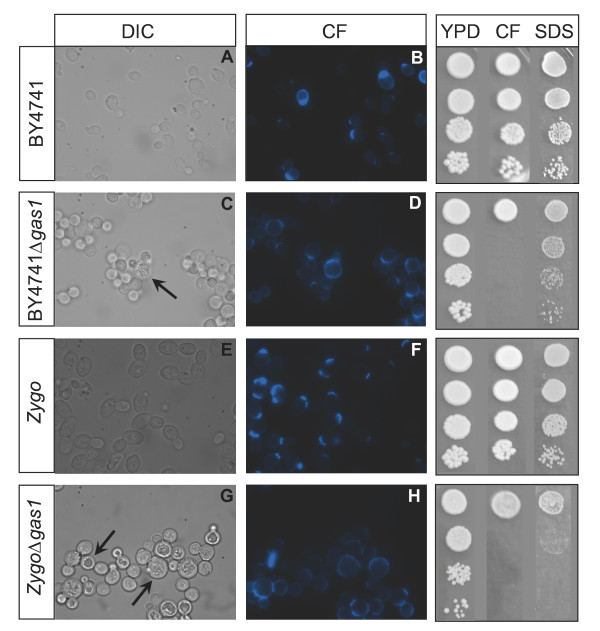
**Morphology of *Z. bailii *and *S. cerevisiae *wild type and *gas1 *null mutant strains and their sensitivity to Calcofluor White and SDS**. Cellular morphology: From A to H: dicroic and fluorescence microscope images of *Z. bailii *and *S. cerevisiae *wild type and *gas*1 null mutant strains. Cells were grown in minimal medium and harvested in stationary phase of growth. Arrows indicate granules and enlarged vacuoles in mutant cells (panels C and G). Calcofluor White and SDS sensitivity of *S. cerevisiae *(BY4741) and *Z. bailii *(*Zygo*) *gas*1 null mutants: 4 μl of cultures of the different strains (BY4741, BY4741 Δ*gas*1, *Zygo *and *Zygo *Δ*gas*1) were spotted starting with OD (660 nm) = 1 and then in 3 serial solution 1:10 (2,3,4) on rich solid medium (YPD) and on the same medium added with 12.5 μg ml^-1 ^of CFW or 50 μg ml^-1 ^SDS. Pictures were captured after 5 days of incubation at 30°C.

The *GAS*1 gene inactivation leads to different peculiar phenotypes in *S. cerevisiae *that were widely characterized [10]. We therefore performed a morphological and physiological characterization of the obtained *Z. bailii *Δ*gas*1 mutant in analogy to what reported in literature for the model yeast. First, some morphological traits of the *Sc *Δ*gas*1 mutant are visible both by light and by fluorescence microscopy after Calcofluor White staining. It was reported for *S. cerevisiae *(both haploid and diploid) mutants a rounded shape and higher cell diameter if compared to wild type cells, especially in the stationary phase [10]. We performed the analysis of the *Zb*Δ*gas*1 and wild type strains in parallel to the *S. cerevisiae *BY4741 Δ*gas*1 and wt strains. Microscopy images are shown in Figure 3. It is possible to see the typical rounded phenotype also for the *Zb*Δ*gas*1 (panels C and G). Moreover, like in *S. cerevisiae*, intracellular granules and altered vacuoles are also visible (indicated by arrows, panels C and G). The altered shape is also reflected by cellular volumes, being higher in the mutants, as assessed by coulter counter measurements in cell populations growing in minimal or in rich medium; like for the model yeast, differences are more pronounced in stationary phase, were the mutant cell volume is 1.6 fold larger in respect to the wild type cells (data not shown). Another feature described for the *Sc*Δ*gas*1 mutant is the appearance of large cellular aggregates, still permanent after repeated sonication cycles [10]. This feature is also conserved in the *Z. bailii *mutant, as it is visible from the microscopy images of sonicated cells (Figure 3, panels C, D, G and H). The morphological alterations described are likely a consequence of the altered structure and composition of the mutant cell wall: it was reported for *S. cerevisiae *a lowered glucans content together with a marked increase in the chitin and mannoproteins content [15]. Moreover, an altered distribution of the cell wall components in the mother and daughter cell have been reported [10]. Our analyses confirmed these alterations also for the *Z. bailii *mutant: overall Calcofluor staining, in fact, was more intense in Δ*gas*1 than in wild type strain (Figure 3, panels D and H), indicating a higher chitin content in the mutant. The chitin seems to be delocalized throughout the entire cell wall in the mutant, while in the wild type it is more concentrated at the budding ring and at the bud scars. Moreover, in the mutant the cellular buds are also Calcofluor-stained, in contrast to the wild type. It was proposed that the increase in chitin levels and cross-links with β-1,6-glucosyl mannoproteins may be the effect of the activation of a compensatory mechanism in mutants characterized by defects in the cell wall architecture, like the Δ*gas*1 mutants. The aim of this response would be to prevent excessive loss of mannoproteins as a consequence of the inability to cross-link the β-1,6-glucosyl mannoproteins and the β-1,3-glucans, a process in which Gas1p plays a central role [15]. It was also hypothesized that the loss of Gas1p may cause a weakening of the cell wall of the emerging bud. The yeast cell could, in response to this weakening, target the accumulation of chitin at the bud surface in order to protect it from lyses [16]. Alteration of the processes directly or indirectly linked to budding leads to the appearance of a relative high fraction, in the cell population, of multibudded cells (the so-called "Mickey Mouse" phenotype) (figure 3). A similar phenotype was also visible in *Zb*Δ*gas*1 mutants (Figure 3).

The altered cell wall structure renders the deleted bakers' yeast strain more sensitive to sodium dodecyl sulphate (SDS) and to Calcofluor White (CFW), as reported in literature for the W303 background [17]. Here we confirm by spot assays the sensitivity to both agents of the *Sc*Δ*gas*1 mutant, background BY4741 (Figure 3, upper right panels), and as well of the *Zb*Δ*gas*1 mutant (Figure 3, lower right panels).

Finally, to verify the functionality of the *GAS*1 homologue of *Z. bailii *in *S. cerevisiae *and the similarity of its biological role, the *ZbGAS*1 gene was expressed into the *GAS*1-deficient *S. cerevisiae *strain, resulting in wild type phenotype restoration either in respect to the morphological traits as well as for the chitin distribution (data not shown). Moreover, to verify the functionality of the *S. cerevisiae GAS*1 in *Z. bailii*, the *ScGAS*1 gene was expressed into the *GAS*1-deficient *Z. bailii *strain, also in this case restoring the native phenotype (data not shown).

Overall morphological and physiological data strongly indicate that the *ZbGAS*1 gene may have a similar role, with respect to the *ScGAS*1 homologous, in the assessment of the cell wall structure and, hence, their inactivation might have similar effects in the two yeasts in respect to secretion abilities.

### Supersecretive phenotype of *Δgas*1 strains

**Figure 4:**
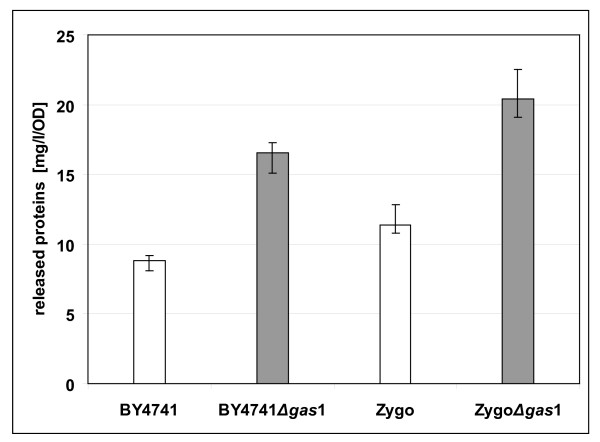
**Total proteins released in the culture medium**. Total proteins released in the culture medium of batch cultures of the indicated wild type and *Δgas*1 strains of *S. cerevisiae *and *Z. bailii*. Yeasts were grown in shake flasks in minimal YNB-based medium with 5% glucose. Cells were inoculated at 0.1 OD and harvested after 72 hours of cultivation. Amount of released proteins (in mg l^-1 ^of culture broth) were normalized for OD values.

As already mentioned (see Introduction), the most interesting phenotype of Δ*gas*1 mutants reported in literature is the supersecretive property. Besides the increased secretion of heterologous proteins, which appears to be dependent on the individual protein [12], the levels of total secreted proteins in late-stationary phase cultures of *S. cerevisiae *mutants were two fold higher with respect to the wild-type [7]. We therefore started our evaluation of secretive properties of the *Z. bailii *mutant, in the perspective of a possible application in industrial processes, by determining the amount of total proteins released in the growth medium during batch cultivation with respect to the wild type strain. *Z. bailii *and *S. cerevisiae *wild type and Δ*gas*1 respective strains were grown in shake flasks in minimal YNB-based medium containing 5% glucose. Cells were harvested at different times of cultivation and the fermentation broths were assayed. At least triplicate experiments were performed on three independent transformants, and Figure 4 shows the mean data obtained reported as mg of total proteins secreted per litre of the culture broth, normalised for OD values. The values refers to 72 h of cultivation, but it was possible to observe a trend leading to this result starting at the end of exponential phase and becoming more pronounced in stationary phase of growth.

The data clearly underline the overall higher secretive capacity of *Z. bailii *if compared to *S. cerevisiae*, in accordance to heterologous proteins secretion data already reported [3]. Moreover, they indicate a significant improvement in the secretive capability of the *Z. bailii *mutant, being nearly 80% more than the wild type in terms of total endogenous protein, similarly to the *S. cerevisiae *mutant.

### Overexpression of CRL lipase, XPR2 protease and hIL-1*β *in *Z. bailii Δgas*1 strains

With the intention to evaluate the potential effects of cell wall engineering and to test the secretive performance in the presence of an heterologous protein, three model proteins were tested: a fungal lipase from *Candida rugosa *(CRL1), a fungal protease from *Yarrowia lipolytica *(XPR2) and the human interleukin 1β (hIL-1β).

**Figure 5:**
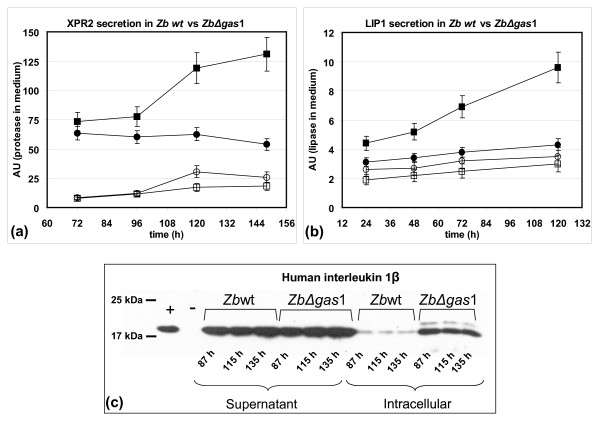
**Secretion levels of yeast protease, lipase and rhIL-1*β *in *Z. bailii and ZbΔgas*1**. (a) Secretion levels of protease in *Z. bailii *and *Zb*Δ*gas*1 grown in min media with 5% glucose. Open circles and open squares indicate wild type and Δ*gas*1 mutant transformed with control plasmid. Solid circles and solid squares indicate wild type and Δ*gas*1 mutant transformed with plasmid with *XPR2 *gene. (b) Secretion levels of lipase in *Z. bailii *and *Zb*Δ*gas*1 grown in min media with 5% glucose. Open circles and open squares indicate wild type and Δ*gas*1 mutant transformed with control plasmid. Solid circles and solid squares indicate wild type and Δ*gas*1 mutant transformed with plasmid with *LIP1 *gene. (c) Western blots for rhIL-1β of protein samples from supernatants and cell extracts of *Z. bailii *and *Zb*Δ*gas*1 transformants grown in YPD media with 5% glucose, inoculated at OD660 = 0,1. From left to right: (A) rhIL-1β; (B) negative control *Z. bailii*[pZ_5_]; (C) (D) (E) (I) (L) (M) *Z. bailii *[pZ_5_*Kl*rhIL-1β]; (F) (G) (H) (N) (O) (P) *Zb*Δ*gas*1 [pZ_5_*Kl*rhIL-1β]. Time and sample types are indicated in the picture.

Four clones of *Z. bailii *Δ*gas*1 [pZLN022*XPR*2] and four clones of the parental strain *Z. bailii *[pZLN022*XPR*2], together with control clones harbouring the respective empty plasmid were pre-cultivated for 16 hours in shake flasks using YPD medium, and then transferred in shake flasks in minimal YNB-based medium containing 5% glucose. The amount of secreted recombinant XPR2 in culture supernatants was tested in terms of enzymatic activity after 72, 96, 120 and 148 h with the Azocoll method (see methods for detail). The *Zb*Δ*gas*1 transformants showed a relevant increase in specific activity levels since 72 h (+ 25% at 72 h, more than two-fold at 148 h, see Figure 5a), in respect to wild type transformants.

Similarly, three clones of *Z. bailii *Δ*gas*1 [pZLN022*LIP*1] and three clones of the parental strain *Z. bailii *[pZLN022*LIP*1], together with control clones harbouring the respective empty plasmid were pre-cultivated for 16 hours in shake flasks using YPD medium, and then transferred in shake flasks in minimal YNB-based medium containing 5% glucose. The amount of secreted recombinant LIP1 in culture supernatants was tested in terms of enzymatic activity after 24, 48, 72 and 120 h using the hydrolysis of *p*-nitrophenylesters method (see methods for detail). Also for this second heterologous product the Δ*gas*1 transformants showed a relevant increase in specific activity levels since 24 h (+35% at 24 h, more than two-fold at 120 h, see Figure 5b), in respect to wild type transformants.

Finally, three clones of *Z. bailii *Δ*gas*1 [pZ_5_*Kl*IL-1β] and three clones of the parental strain *Z. bailii *[pZ_5_*Kl*IL-1β], together with control clones harbouring the respective empty plasmid were tested for secretion level of the human interleukin, since the coding sequence was functionally linked to the signal peptide of the K1 killer toxin from *K. lactis*. The independent transformants were pre-cultivated and then cultured in shake flasks containing rich medium (YPD, 5% w/v glucose). Samples were collected at the indicated times (see Figure 5c), cells were harvested and both the supernatants and the cell protein extracts of said samples were loaded on a polyacrylamide gel. The data obtained by Western blot (see Figure 5c) performed on the supernatant showed a well represented band corresponding to the recombinant protein for both *Z. bailii *and *Z. bailii *Δ*gas*1 samples, but differently from the case of recombinant fungal enzymes, no relevant differences in favour of the deleted yeast are appreciable. Remarkably, the signal corresponding to the protein retained intracellularly is more intense in *gas1 *null mutant than the signal obtained from *Z. bailii *parental strain, indicating an incomplete secretion of this protein.

## Discussions

Cell wall engineering can improve the release of heterologous proteins from yeasts [18]. Generally speaking, *GAS*1, which mediates the crosslinking of cell wall components, is directly involved in defining the permeability of the cell. Cloning of the unknown *Z. bailii GAS*1 homologue by PCR was only possible starting from highly conserved internal regions, identified upon alignment of the *GAS*1 homologues of three yeast species. The 5' and 3' ends were subsequently amplified using a Genome Walker library. With this approach, the full-length *Z. bailii GAS*1 homologue could be amplified and sequenced. The cloned gene was expressed in the *GAS*1-deficient *S. cerevisiae *strain and reversion of the phenotype proved that this homologue is not only structurally but also functionally similar to the *S. cerevisiae *gene. Moreover, also the *ScGAS*1 gene complemented the *Z. bailii *mutant. Disruption of *GAS*1 in *Z. bailii *revealed its influence on the cell wall morphology. The *GAS*1 null mutants of *Z. bailii *showed the same phenotype reported for *S. cerevisiae *[10], with enlarged cells often showing granular cytoplasm, an alterated chitin distribution, two buds (Mickey Mouse-like appearance) and big vacuoles. The vacuole is known to be a dynamic organelle whose morphology is highly responsive to different extracellular and intracellular stimuli/stressors [19]. In the *GAS*1 null mutant, it can be therefore speculated that the generation of big vacuoles is related to stressful conditions, as already suggested by the altered chitin distribution being a possible defence mechanism against lysis or related to a generalized response to sickness of the cells [10].

Finally, also the sensitivity to SDS and CFW appeared very similar to what observed in the *S. cerevisiae *mutant. A comparison of the different *GAS*1 null phenotype mutants and the functionality of the *Z. bailii GAS*1 homologue in *S. cerevisiae *suggest a similarity of the cell wall structure of *Z. bailii *compared with the cell wall structure of *S. cerevisiae*. Considering the secretory pathway of a secreted protein, it was speculated whether the protein becomes retarded in the cell wall after release from the plasma membrane. The disruption of *GAS*1 in *Z. bailii *first of all confirmed a general phenomenon of protein release in respect to the wild type strain, as demonstrated by the higher accumulation of total protein in the growing medium of the deleted strain when compared to the parental strain. Moreover, such as compromised cell wall permeability in the production strains for XPR2 and LIP1 does lead to a clear improvement in the secretion of the respective proteins. In the case of h IL-1β the enhancement of product secretion was not so evident, and difficult to interpret in respect to the higher accumulation of intracellular product. It could be speculated that this can be related to the nature or to the source of the protein, but too few information are available at the moment to further comment in this direction. What seems to be true is unfortunately what we very often experience for recombinant protein production, which is the protein and protein-host dependence. In fact, also in *P. pastoris *two out of three heterologous proteins resulted more secreted in the mutant, as for *Z. bailii*, and when we tested the same protein, in particular the IL-1β secretion in the *S. cerevisiae *Δ*gas*1 mutant (data not shown), we did not register any improvement.

## Methods

### Yeast strains, media and transformation

The *S. cerevisiae *strains used in this study were the auxotrophic haploid BY4741 (*Mat a*, *his3*Δ*1*, *leu2*Δ*0*, *met5*Δ*0*, *ura3*Δ*0*) and the relative null mutant in *GAS*1 sequence, called in this study, BY4741 Δ*gas*1 (*Mat a*, *his3*Δ*1*, *leu2*Δ*0*, *met5*Δ*0*, *ura3*Δ*0*, YMR307*::kanMX4*). Both strains are commercially available from Euroscarf collection http://web.uni-frankfurt.de/fb15/mikro/euroscarf/col_index.html. The *Z. bailii *wild type strain is ATCC60483. Yeast cultures were grown in YP medium (10 g l^-1 ^yeast extract, 20 g l^-1 ^peptone) with 2% or 5% w/v glucose (D) or fructose (F) as carbon source. Where needed, NAT (80 mg l^-1^) or G418 (500 mg l^-1 ^for *S. cerevisiae *and 200 mg l^-1 ^for *Z. bailii*) or hygromicine (100 mg l^-1 ^for *Z. bailii*) were added to the media. Alternatively, yeast cultures were grown in minimal synthetic medium (0.67% w v^-1 ^YNB Biolife without amino acids) with 2% or 5% w v^-1 ^glucose as carbon source. When required, supplements such as histidine, leucine, methionine and uracil were added to a final concentration of 50 mg l^-1^. Media for plates were solidified by addition of agar (Biolife, Milan, Italy) to 1.5% w v^-1^. Shake flask experiments were carried out with 50 ml of medium in 250 ml shake flasks on a shaker at 30°C with 160 rpm. For sensitivity tests in Petri dishes 12.5 μg ml^-1 ^of CFW or 50 μg ml^-1 ^SDS were added to solid media. Yeast transformation was performed using the Lithium Acetate/ssDNA method as described [20] and slightly adapted for *Z. bailii*, as described [3].

### Genes amplifications, deletion cassettes and expression plasmid construction

PCR amplifications were made from yeasts genomic DNA, prepared as described in [21]. Amplifications were performed by the Pwo DNA Polymerase (Roche).

The amplification of the *ZbGAS*1 gene was as follows: the primers *GAS*1int_fw: 5'-AGT TGT TCC AGA GAT ATT CCA TAC CTC AA-3' and *GAS*1int_rev: 5'-GCA GAA CCG CTG AAG CTA CAG T-3' designed on internal conserved region of the available sequences from other yeasts were firstly used to amplify an internal region of 1137 bp of the *Z. bailii *gene with the following program: 94°C 5 min; (94°C 15 s, 57°C 30 s, 72°C 1 min) × 25; 72°C 7 min; 4°C ∞. After that, the designed primers *ZbGAS*1int_fw: 5'-GGA CGA TAA GGT TGA CGA AGA-3' and *ZbGAS*1int_rev 5'-TGC TGT GGT CCA AAG TTG TG-3' were used for the amplification (program: 7 cycles: 94°C 2 min, 72°C 3 min; 32 cycles: 94°C 2 min, 67°C 3 min; 67°C 4 min; 4°C ∞) of the terminal ends of *ZbGAS*1 utilizing a *Z. bailii *Genome Walker library constructed using the Universal Genome Walker kit (Clontech) following the manufacturers instructions. From the obtained and reconstructed sequence the final primers were established and used for the *ZbGAS*1 1710 bp full length coding sequence amplification: *ZbGAS*1_fw: 5'-ACT AAT GTT ATT CCA GGC GTT TTC G-3' and *ZbGAS*1real_rev: 5'-AAA TCA AGC CAA AGC AAA TCC AGC A-3' (program: 94°C 5 min; (94°C 20 s, 58°C 30 s, 72°C 2 min) × 30; 72°C 10 min; 4°C ∞). The DNA sequence of *ScGAS*1 was PCR amplified with the following primers: *ScGAS*1fw: 5'-ACA ATG TTG TTT AAA TCC CTT TCA AAG TTA GCA A-3' and *ScGAS*1rev: 5'-TTT TTA AAC CAA AGC AAA ACC GAC ACC AG-3' (program: 94°C 5 min; (94°C 20 s, 60.5°C 30 s, 72°C 2 min) × 30; 72°C 10 min; 4°C ∞), and the DNA sequence of *YlXPR*2 was PCR amplified with the following primers: *XPR*2_fwd: 5'-ACA ATG AAG CTC GCT ACC GCC TTT A-3' and *XPR*2_rev: 5'-TGC CTA AAT GCC AAC ACC GTT GTA G-3' (program: 94°C 4 min; (94°C 15 s, 62.5°C 30 s, 72°C 2 min) × 30; 72°C 7 min; 4°C ∞). All the unique fragments obtained were sub-cloned in the vector pSTblue-1 utilising the Perfectly Blunt^® ^Cloning Kit (Novagene) and sequenced. The resulting respective plasmids utilised in this work were named as follows: pSTb*Zb*GAS1int, pSTb*Zb*GAS1, pSTb*Sc*GAS1 and pSTb*Yl*XPR2.

For the construction of the *ZbGAS*1 deletion cassettes, the Kan^R ^or the hph^R ^cassette, derived from plasmid pFA6-KanMX4 [22] and pAG26 [23] and conferring resistance to G418 and hygromycin respectively, were bluntended excised and inserted in the pSTb*Zb*GAS1int opened *Bst*EII *Bgl*II, bluntended and dephosphorylated, resulting in the plasmids pSTb*Zb*GAS1ΔK and pSTb*Zb*GAS1ΔH. The resistance cassettes resulted in both cases counter-clockwise inserted. The *Z. bailii *wild type cells were transformed with the respective deletion cassettes obtained by cutting the two described plasmids in the *Acc*I and *Sna*BI sites. The effective deletion was PCR checked by using the following couples of primers: ZbG1int2_fw 5'- TGC AGA AGT TAC AGA CCA ATG TTG T -3' and and ZbG1int2_rev 5'-AAG ATA GAG AAG TGC TCT TGG CA -3' (see Figure 2) or ZbG1int2_fw and Hph_fw 5'- ATA TGA AAA AGC CTG AAC TCA CCG AC -3' or Kan_fw 5'- ATG GGT AAG GAA AAG ACT CAC GTT -3' (not shown) in order to verify the presence of the cassettes in the desired locus.

For complementing the yeasts *GAS*1 deletion, pZ_5_(-Nco)*ScGAS1 *and pYX022*ZbGAS1 *were constructed. pZ_5_(-Nco)*ScGAS1 *was obtained from pZ5(-Nco) opened *Sac*I/*Eco*RV with the insertion of *ScGAS1*, from pSTb*ScGAS1 *cut with *Sac*I/*Sna*BI. The pZ_5_(-Nco) derives from pZ_3 _[3] where at the *Kpn*I site the *Kan*^R ^cassette was substituted from the NAT^R ^cassette obtained from vector pAG25 [23] by cutting *Pvu*II/*Sac*I/blunt (obtaining the pZ_5 _plasmid): finally this plasmid was *Eco*RI/*Bam*HI cut and bluntended and reclosed. pYX022*ZbGAS1 *was obtained with the insertion of *ZbGAS*1 from pSTb*ZbGAS1 *into pYX022 (R&D Systems, Wiesbaden, Germany) both *Eco*RI cut.

For heterologous protein expression the plasmids pZLN022*XPR2*, pZLN022*LIP1 *and pZ_5_*Kl*hIL-1β were constructed. The first two integrative plasmids derives from pZLN022 (manuscript in preparation), obtained from pYX022 opened *Dra*III blunt/*Spe*I blunt (to exclude *HIS*3) with the insertion of *ZbLEU*2 cut from pSTb*ZbLEU*2 with *Eco*RI blunt; in *Kpn*I blunt site the NAT^R ^cassette was inserted, obtained from vector pAG25 [23] by cutting *Pvu*II/*Sac*I/blunt. The pSTb*ZbLEU*2 was obtained by sub-cloning *ZbLEU*2 amplified by PCR from *Z. bailii *genomic DNA (manuscript in preparation) in pSTBlue (Novagen) cut by *Eco*RV. For pZLN022*XPR2 *construction, the pZLN022 was *Aat*II/*Nhe*I cut, and the *XPR2 *from pSTb*XPR2 *was inserted. For pZLN022*LIP1 *construction, the pZLN022 was *Aat*II blunt/*Nhe*I blunt opened and the LIP1 was inserted by *Bam*HI blunt/*Nsi*I blunt excision from the pGAP*sLIP *[24]. For rh interleukin the pZ_5_*Kl*hIL-1β was built: it was obtained from pZ_5 _opened with *Eco*RI-blunt, with the insertion of hIL-1β in frame with *Kl *signal sequence from vector pKSSPI/3 [25] cut *Eco*RI blunt/*Xba*I blunt.

DNA manipulations, transformation and cultivation of *Escherichia coli *(DH5αF' (φ80dlacZΔM15, Δ (lacZYA-argF), U169, deo, rec1, end1, sup44, λ, THI-1, gyrA96, relA1) and Novablue Competent Cells (Novagene) were performed following standard protocols [26]. All the restriction and modification enzymes used were from New England Biolabs (Hitchin, Herts, UK) or from Roche Diagnostics (Mannheim, Germany).

### Calcofluor staining and fluorescence microscopy

Cells were harvested, washed in PBS and resuspended in 1 mg ml^-1 ^Calcofluor White solution, incubated 10-30 min at room temperature, washed again twice in PBS. Fluorescence microscope images were taken by a Nikon Eclipse 90i microscope using the Metamorph software version 2.2 (Nikon), using emission filter UV-1A.

### Quantification of total released proteins

Cultures were harvested and centrifuged at maximal speed, then total proteins in the supernatants were ethanol-precipitated. The amount of proteins was determined by the dye-binding method of Bradford (Quick Start Bradford Dye Reagent - BIORAD), using BSA as standard. All the data were normalized by OD, after verification of the linear correlation between OD and dry weight as a measure of biomass.

### Quantification of secreted protease

Cultures were harvested and centrifuged at maximal speed, then total proteins in the supernatants were separated and stored at -20°C until analysis. The amount of protease secreted in the medium was determined by the Azocoll assay (Azocoll - Calbiochem), using commercial protease as positive control. Azocoll assays were performed according to [27] with some modifications. Reaction mixtures contained 15 mg ml^-1 ^Azocoll, 50 mM Tris/HCl (pH 7.6), 150 mM NaCl and 5 mM CaCl_2_. Proteolytic activities were measured by absorbance changes at 490 nm (Shimadzu UV 1601) after incubation for 18 h at 30°C.

### Quantification of secreted lipase

Cultures were harvested and centrifuged at maximal speed, then the supernatants were separated and stored at -20°C until analysis. The amount of lipase secreted in the medium was determined according to [28] with some modifications. Reaction mixtures contained: (A) 60 mg of *p*-nitrophenyl palmitate (Sigma Aldrich) in 20 ml of isopropanol (Sigma Aldrich), (B) 1 g Triton X-100 (Sigma Aldrich) and 0.2 g of Arabic gum (Sigma Aldrich) in 200 ml Tris/HCl 0.1 M (pH 7.5). To obtain the mixture (C) add slowly 1 part of A solution to 9 part of B solution under continuous agitation. Add 9 part of (C) solution to one part of the enzyme containing solution (*i.e*. the supernatant). Lipase activities were measured by absorbance changes at 410 nm (Shimadzu UV 1601) and the amount of enzyme is proportional to OD according to the relation U/m = 0.667 × OD_410 nm _(if OD_410 nm _< 0.5).

### Quantification of interleukin

Human IL-1β were analysed by Western blot. Independent transformants were cultured in shake flasks in minimal or rich medium and during the kinetics samples were collected. Cells were harvested (a culture volume corresponding to 10^8 ^cells) by centrifugation. One volume of 2×Laemmli buffer [29] was added to the supernatants of said samples, they were boiled for 3-5 min and stored at -20°C until loading, or loaded directly on a polyacrylamide gel. For the analysis of intracellular production, crude extracts were prepared from 10^8 ^cells by following the trichloroacetic acid protocol [30] and resuspending the final protein extract in 150 μl of Laemmli buffer. Samples were then loaded on standard polyacrylamide gels (sodium dodecyl sulfate-polyacrylamide gel electrophoresis, final concentration of the separating gel: 15%); after separation, proteins were blotted to nitrocellulose membranes and immunodecorated. An anti-IL-1β rabbit polyclonal antibody (IL-1β(H-153), Santa Cruz Biotechnology, USA; cat. no. sc-7884; dilution 1:200) was used for the interleukin immunodecoration. An anti-rabbit IgG horseradish peroxidase-conjugated (Amersham Pharmacia Biotech, UK cat no. NA934; dilution 1:10.000) secondary antibody was used. For the interleukin detection, as a positive control, the human recombinant IL-1β from *E. coli *(Roche cat. no. 1 457 756) was always loaded in parallel on the polyacrylamide gels. The proteins were visualised using the ECL Western blotting system (Amersham Biosciences, UK), according to the manufacturer's instructions.

## Competing interests

The authors declare that they have no competing interests.

## Authors' contributions

PB and DP initiated and coordinated the project. LD was responsible for *Zb*GAS1 gene identification by PCR and its deletion. LR analyzed the mutant phenotype with CFW and SDS. LR and SP performed the batch cultivation, analyzed the secretory phenotype in null mutant and performed the experiment with heterologous proteins. All authors wrote the paper and approved the final version of the manuscript.

## Supplementary Material

Additional file 1 Cl**ustalw_ntd**. Nucleotidic sequence of the *Zb*GAS1 gene and alignment with the homologue *Sc*GAS1 (entry: YMR307W). Grey coloring represent nucleotidic identity. Alignment was performed with ClustalW software http://www.ebi.ac.uk/clustalw and graphical editing was made using Jalview software [31]. Click here for file
