# Supplementary material for: Cloning of the Zygosaccharomyces bailii GAS1 homologue and effect of cell wall engineering on protein secretory phenotype
Source: Microb Cell Fact. 2010 Jan 26;9:7. doi: 10.1186/1475-2859-9-7 (PMC2825207; doi:10.1186/1475-2859-9-7)
Supplement: Additional file 1 — Clustalw_ntd. Nucleotidic sequence of the ZbGAS1 gene and alignment with the homologue ScGAS1 (entry: YMR307W). Grey coloring represent nucleotidic identity. Alignment was performed with ClustalW software http://www.ebi.ac.uk/clustalw and graphical editing was made using Jalview software [31]. [file 1475-2859-9-7-S1.PDF]

```
ZbGAS1 ATGTTATTCCAGGCGTTTTCGACCCTCGCACTTGGTGCACTATACGCTGCCTCCGGTGC 59
ScGAS1 ATGTTGTTTAAATCCCTTTCAAAGTTAGCAACCGCTGC------TGCTTTTTTTGCTGG 53

ZbGAS1 AGTTGCAGCCAGTAGCAGCAGTGCTACCAAACTTCCAGCAATTGAAGTTGCTGGTAACA 118
ScGAS1 CGTCGCAAC------------TGCGGACGATGTTCCAGCGATTGAAGTTGTTGGTAATA 100

ZbGAS1 AGTTCTTCTACTCGAACAACGGTTCTCAATTTTACATCAAAGGTGTTGCATACCAGGCC 177
ScGAS1 AGTTTTTCTACTCCAACAACGGTAGTCAGTTCTACATAAGAGGTGTTGCTTATCAGGCT 159

ZbGAS1 GACACGGCTAACTCATCTTCTGATGACAGCATCGACGATCCATTGGCCGACTACTCCAA 236
ScGAS1 GATACCGCTAATGAAACTAGCGGATCTACTGTCAACGATCCTTTGGCCAATTATGAGAG 218

ZbGAS1 ATGTTCGAGAGATATTCCATATTTGCAGAAGTTACAGACCAATGTTGTTCGTGTCTACG 295
ScGAS1 TTGTTCCAGAGATATTCCATACCTCAAAAAATTGAACACAAATGTTATCCGTGTCTACG 277

ZbGAS1 CAGTTAACACAACTTTGGACCACAGCAAGTGTATGGAAGCTCTAGCTGACGCTGGTATT 354
ScGAS1 CTATCAATACCACTCTAGATCACTCCGAATGTATGAAGGCTTTGAATGATGCTGACATC 336

ZbGAS1 TACGTCATTGCAGACTTGTCCACCCCAGCTGACTCTGTTAACAGAAATGACCCTACTTG 413
ScGAS1 TATGTCATCGCTGATTTAGCAGCTCCAGCCACCTCTATCAATAGAGACGATCCAACTTG 395

ZbGAS1 GGATATCGCACTATACCAGCGTTACACTGGCGTGGTGGACGCTTTTGCTAACTACACAA 472
ScGAS1 GACTGTTGACTTGTTCAACAGCTACAAAACCGTTGTTGACACTTTTGCTAATTACACCA 454

ZbGAS1 ACGTGTTGGGTTTCTTTGCCGGTAACGAAGTGACCAACAATGCTAGTAACACTGATGCA 531
ScGAS1 ACGTTTTGGGTTTCTTCGCCGGTAATGAAGTTACTAACAATTACACCAACACAGATGCA 513

ZbGAS1 TCTGCCTTTGTGAAGGCTGCCGTTAGAGACGTTAAGCAGTACATCAAGGACAAGAAATA 590
ScGAS1 TCTGCTTTCGTGAAGGCAGCTATTAGAGACGTCAGACAATACATCAGCGACAAGAACTA 572

ZbGAS1 CAGAACAATTCCTGTTGGTTACTCCTCCAATGATGACGAGGACACCAGAGTTGCTATGG 649
ScGAS1 CAGAAAAATTCCAGTTGGCTACTCTTCCAATGATGACGAAGATACCAGAGTTAAGATGA 631

ZbGAS1 CTGACTACTTTGCTTGCGGTGACGAAGACCAAAAGGCTGATTTCTACGGTATTAACATG 708
ScGAS1 CTGATTATTTCGCTTGTGGTGATGATGATGTTAAGGCTGATTTTTACGGTATTAATATG 690

ZbGAS1 TACGAATGGTGTGGTGACTCAACCTACCAGAAATCTGGTTACCAGGACAGAACCAATGA 767
ScGAS1 TATGAATGGTGTGGTAAATCTGACTTCAAAACTTCTGGTTATGCTGATAGAACTGCAGA 749

ZbGAS1 CTTCAAGAATTTGTCGATCCCTATCTTCTTCTCTGAGTACGGTTGCAACGCTGTCACTC 826
ScGAS1 ATTCAAAAACTTATCTATTCCTGTTTTCTTCTCTGAATACGGTTGTAACGAAGTAACAC 808

ZbGAS1 CAAGAAAGTTTACCGAGGTTCAGACTCTTTACGGTGATCAGATGACTGATGTGTGGTCC 885
ScGAS1 CAAGACTATTTACTGAGGTTGAAGCCTTGTACGGTTCTAATATGACAGATGTCTGGTCT 867

ZbGAS1 GGTGGTATTGTTTACATGTATTTCGAAGAGACCAACAAGTACGGTTTGGTCAGTATTGA 944
ScGAS1 GGTGGTATCGTATACATGTACTTCGAAGAGACTAACAAATACGGTTTAGTTAGCATCGA 926

ZbGAS1 TGATGATCAGGTCAAGACTTTGACTGACTTCGACAACTTGTCGAGCCAGATGGCCAAGA 1003
ScGAS1 TGGTAATGATGTTAAAACTTTGGATGACTTCAACAACTATTCTTCTGAAATCAACAAAA 985

ZbGAS1 TCTCTCCAAGTGCTGCCAAGAACTCTTCTTACACTGCCAAGAGCACTTCTCTATCTTGC 1062
ScGAS1 TATCACCAACTTCCGCCAACACAAAGTCTTACAGTGCAACAACAAGTGATGTTGCTTGT 1044

ZbGAS1 CCAGCCACCGGCAAGTACTGGAAAGCTAACACCAAATTGCCACCTACCCCAAGCAAGGA 1121
ScGAS1 CCAGCTACTGGTAAGTACTGGTCCGCTGCAACAGAATTACCACCAACTCCAAACGGAGG 1103

ZbGAS1 CCTCTGTACCTGTATGGAAGACTCCTTGTCCTGTGTTGTGGACGATAAGGTTGACGAAG 1180
ScGAS1 CTTGTGTTCATGTATGAATGCAGCCAATAGTTGTGTTGTTTCCGATGACGTTGATTCTG 1162

ZbGAS1 ATGACTACAGTGATTTGTTCAGTTACATCTGCTCGAAGGTGGACTGTTCTGGTATTACC 1239
ScGAS1 ATGATTACGAAACCTTATTTAACTGGATCTGTAATGAAGTCGACTGTAGCGGTATTTCA 1221

ZbGAS1 GCCAACGGTACTAGTGGTAAATATGGTTCTTACTCTTTCTGCTCTGCCAAGGAGCAGCT 1298
ScGAS1 GCAAACGGTACCGCCGGTAAGTATGGTGCTTACTCTTTCTGTACACCAAAGGAACAGCT 1280

ZbGAS1 ATCCTTTGTCATGAACTTGTATTACGAGAAGAACGGTGGTAGCAAGTCTGACTGTAGCT 1357
ScGAS1 ATCTTTCGTTATGAATTTGTACTACGAGAAGAGTGGTGGTAGCAAATCTGACTGTAGCT 1339

ZbGAS1 TCAGTGGTTCTGCCAGCTTGAAGTCTGCCACCACCAAGTCTGCCTGTTCCTCTGCTTTG 1416
ScGAS1 TCAGCGGTTCTGCCACTCTACAAACTGCCACCACGCAAGCTAGTTGCTCCTCCGCTTTG 1398

ZbGAS1 AAGCAGATTGGTAGCTCCGGTCTAGGCTCTGCCAGCAAAAGTGTCACTGGTTCCGCCTC 1475
ScGAS1 AAAGAGATTGGTAGTATGGGTACCAACTCTGCATCAGGTAGTGT---TGATTTGGGTTC 1454

ZbGAS1 TGGCTCCTCCTCTGGCTCATCTTCTGCTTCTGGTTCTTCTGAATCTTCCTCTGGCAAGG 1534
ScGAS1 CGGAACTGAATCCAGTAC---TGCCTCTTCTAACGCTTCGGGGTCTTCTTCCAAGTCTA 1510

ZbGAS1 GTTCTAGCTCCAGTAAGGGTGGTGACTCTTCCTCTAGTTCCAAGAAGAGCTCAAGTACA 1593
ScGAS1 ACTCCGGCTCTTCTGGTTCTTCCAGTTCTTCTTCTTCTTCAG---CTTCATCTTCA 1566

ZbGAS1 GCTAGCACCAAGGAAAAGAACTTTGCTGCCGCTTCCAAGGCTAGTCTGTCTCAGGTAAT 1652
ScGAS1 TCTTCTAGCAAG---AAGAATGCTGCCACCAACGTTAAAGCTAACTTAGCACAAGTGGT 1622

ZbGAS1 CCTATCCTTCGTGGCCACTTTGGGTGTGGTCGCCGGTGCTGGATTTGCTTTGGCTTGA  1710
ScGAS1 CTTTACCTCCATCATTTCCTTATCCATTGCCGCTGGTGTCGGTTTTGCTTTGGTTTAA  1680
```
